# Suggestions in Reference to the Cumming’s Patent

**Published:** 1872-06

**Authors:** S. J. Cobb


### CORRESPONDENCE.

### Suggestions in Reference to the Cumming's Patent.

S. J. COBB.

*Publishers Dental Register:*

DEAR SIRS: A few days before receiving your last issue I read a notice from Bacon & Co. that they had gained the suit appealed by Gardner to the Supreme Court of the United States. Somehow or other I have had my misgivings about this suit ever since the case was taken up. I did not, and do not now, think that the validity of this Cumming's patent should have been tried until the Goodyear patent expired; but from some cause or other it was taken up, and put through in such a way and time as to give Bacon & Co. just such a decision as they desired. While I know nothing of the points presented on either side in this case, I would suggest the propriety of waiting until we can find out how the facts were gotten up and presented to the court. If all has been done that we can do, and all is right, I have nothing more to say, but if otherwise, I, for one, am in favor of taking up another case, which will be on the Cumming's patent alone. To do this we must all get together and work as one man, and to do this effectually we must have a national meeting, which, by the by, I think would be a very good thing for us anyhow. An opportunity of this sort would enable us to get up one of the largest meetings that we have ever had. There would be no trouble in getting the North, South, East and West together on such an occasion, especially as our national societies (so called) have almost run down just at this time. I would like very much to attend a large National Dental Convention; we have never had just such a meeting in this country. The result of such a meeting would be beneficial to the whole profession, to say nothing of the rubber question, which we can not afford to pass over without a thorough investigation. We hope our journals will be able to furnish us full and complete information in regard to the Gardner suit. Surely the dentists in Washington City, and others near by, were sufficienly interested to get the facts in this case, so as to satisfy the profession whether all is right or not. If they should fail to do this, we must have a national committee to see how it was done. While I am not much surprised at the decision reached by the court, I am at a loss to know how it is that one can get a patent for a thing that has been in common use for a half or dozen years before a patent is applied for. The very thing that Mr. Cumming's claims as his invention was in use at least a dozen years before he applied for a patent. His patent, as far as the method is concerned, which is just what he claims, covers the ground of all molded plates. Cheoplastic, which has been made as long ago as some of us can remember, was made precisely as rubber plates are at present, as far as putting up the teeth in wax and the plaster model is concerned. The only difference in making white metal and rubber plates is in pouring and pressing, the metal being poured instead of pressed in the mould. Celluloid plates are made, with the exception of vulcanizing, just exactly as rubber. So you see Mr. Bacon & Co. is going to take every thing from us. If they were entitled to all this, we should quietly submit, but as we all feel and know to the contrary, we should bestir ourselves in establishing these facts. Without preferring charges against anybody, I appeal to the dentists of the United States to investigate this Cumming's patent. We should have organized last year, and have been fully prepared, which would have prevented this premature trial.

